# A de novo *KMT2D* mutation in a girl with Kabuki syndrome associated with endocrine symptoms: a case report

**DOI:** 10.1186/s12881-018-0606-9

**Published:** 2018-06-18

**Authors:** Jung-Eun Moon, Su-Jeong Lee, Cheol Woo Ko

**Affiliations:** Department of Pediatrics, Kyungpook National University School of Medicine, Kyungpook National University Children’s Hospital, 807, Hoguk-ro, Buk-gu, Daegu, 41404 Republic of Korea

**Keywords:** Kabuki syndrome, *KMT2D* gene, Growth hormone deficiency, Constitutional delay of puberty

## Abstract

**Background:**

Kabuki syndrome is characterized by distinctive facial features and varying degrees of growth retardation. It leads to malformations in skeletal, urogenital and cardiac structures; moreover, endocrine conditions such as premature thelarche, precocious puberty, growth hormone deficiency, diabetes insipidus, thyroid dysfunction and obesity have been reported. Kabuki syndrome is caused by a heterozygous mutation in the *KMT2D* or *KDM6A* genes.

**Case presentation:**

An 11-year-old girl with the typical facial features of Kabuki syndrome visited our hospital due to her short stature. She was found to have the de novo heterozygous mutation of c.8200C > T, p(Arg2734*) in exon 32 of the *KMT2D* gene and was diagnosed with Kabuki syndrome. The patient also exhibited endocrine abnormalities such as a constitutional delay of puberty, transiently congenial hypothyroidism, obesity and growth hormone deficiency.

**Conclusions:**

This is a case of a mutation in the *KMT2D* gene in a girl with Kabuki syndrome who presented with endocrine symptoms (constitutional delay of puberty, hypothyroidism, obesity and growth hormone deficiency).

**Electronic supplementary material:**

The online version of this article (10.1186/s12881-018-0606-9) contains supplementary material, which is available to authorized users.

## Background

Kabuki syndrome (OMIM#147920), which was first reported in Japan in 1981, is a rare disease with an incidence of 1:32,000 in Japan [1, 2]. At least 589 patients with Kabuki syndrome due to *KDM6A* mutations have been reported [3]. Kabuki syndrome is characterized by distinctive facial features, including long palpebral fissures with eversion of the lateral third of the lower eyelids, arched eyebrows and a short nasal columella with a broad and depressed nasal tip; it also manifests as varying degrees of growth retardation [4]. Kabuki syndrome is a multiple malformation syndrome that is accompanied by skeletal conditions such as cleft palate and cranial abnormalities (including coronal and metopic synostosis), as well as urogenital and cardiac abnormalities [5–7]. Patients with this syndrome also exhibit endocrine system-related conditions such as premature thelarche, precocious puberty, diabetes insipidus, thyroid dysfunction, obesity and growth hormone (GH) deficiency [8–10].

Kabuki syndrome is caused by mutations in the *KMT2D* (NM_003482.3, also known as *MLL2*, *MLL4*) or *KDM6A* (NM_021140.3) genes. A mutation in *KMT2D* is the predominant cause of Kabuki syndrome [7, 11]. *KMT2D* encodes the histone-lysine N-methyltransferase 2D protein, which is essential for early embryonic development and acts as a mono-methyltransferase for histone H3 lysine 4 (H3K4), which in turn is involved in gene regulation, reproduction, organogenesis, and disease [12].

We report the case of a girl with a de novo *KMT2D* mutation who had the typical Kabuki syndrome features as well as endocrine-related symptoms, including a constitutional delay of puberty (CDP), obesity and GH deficiency.

## Case presentation

An 11-year, 7-month-old girl visited the Kyungpook National Children’s Hospital because of a short stature. The patient had been born via vaginal delivery at a gestational age of 38 weeks; she weighed 2.6 kg at birth, and there were no perinatal problems. However, she had an incomplete cleft palate and craniosynostosis at birth. Hearing loss in both ears was detected at the age of 1 month by brainstem-evoked response audiometry during an initial screening test. An ‘inborn errors of metabolism’ workup performed after birth revealed congenital hypothyroidism, for which the patient received levothyroxine at the local hospital until she was 3 years old; normal thyroid function was confirmed after discontinuing the medication.

When she was 1 year old, she was transferred to our hospital because of a developmental delay and for a repair of her incomplete cleft palate and craniosynostosis. Brain computed tomography revealed an auditory defect, and she began wearing hearing aids, as prescribed by an otolaryngologist. The patient underwent skull reconstruction and incomplete cleft palate repair at the Departments of Plastic Surgery and Neurosurgery, respectively. She was scheduled for follow-up at the Department of Pediatric Neurology due to her developmental language delay, but she was lost to follow-up.

**Fig. 1:**
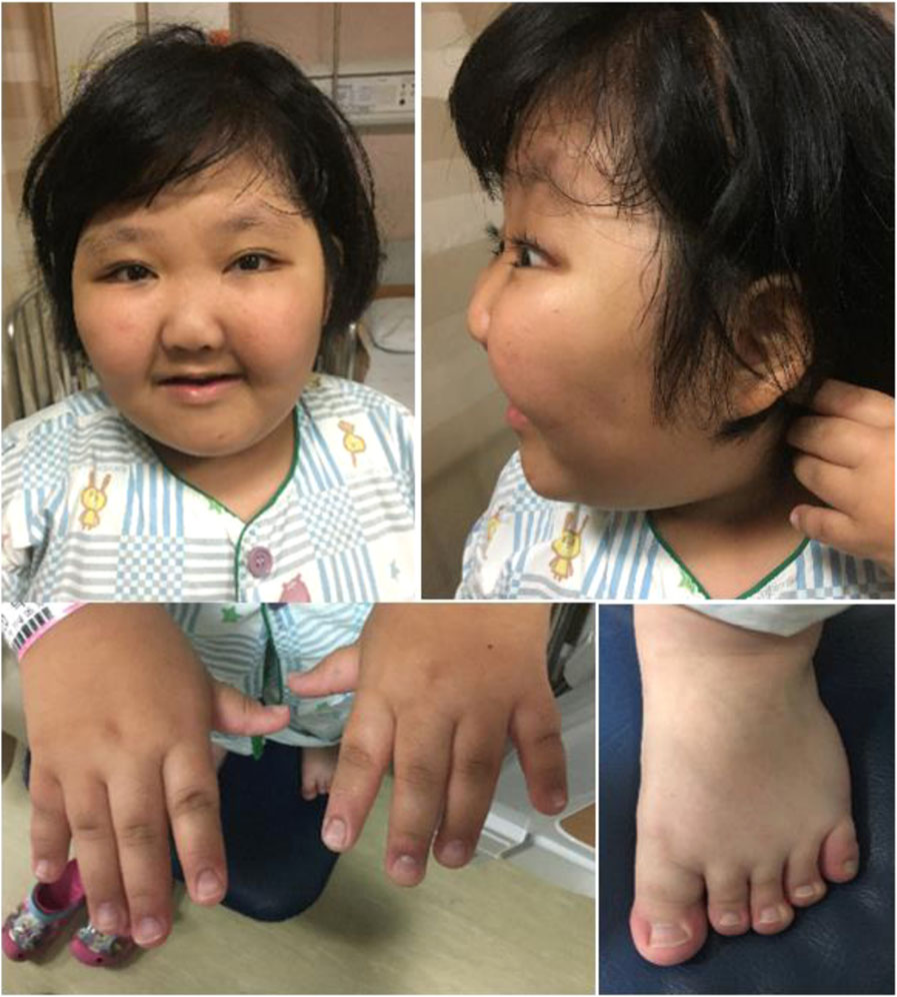
The patient shows the typical facial features of Kabuki syndrome, such as long palpebral fissures, everted lower eyelids, arched eyebrows, a broad nasal root, thin upper and full lower lips, and brachydactyly in both hands and feet

When the patient re-visited our hospital at the age of 11 years and 7 months, she had a height of 124.8 cm (standard deviation score [SDS]: − 3.6), weight of 46 kg (SDS: 0.65 kg), and body mass index (BMI) of 29.53 kg/m^2^ (Z score: 1.89). She had distinctive facial features, including abnormally long openings between the eyelids, arch-shaped eyebrows, a thin upper lip, and large ears (Fig. 1). Moreover, she showed postnatal growth retardation and skeletal anomalies, including an incomplete cleft palate, craniosynostosis, and brachydactyly (Fig. 1). These dysmorphic features and her developmental delay were considered suggestive of Kabuki syndrome. Her karyotype was 46,XX. Her bone age was 8 years and 10 months, which was considered delayed according to the Greulich and Pyle atlas [13]. Based on these characteristic clinical features, mutational analyses for the *KMT2D* and *KDM6A* genes were performed.

The CARE guidelines were followed in reporting this case, and the molecular analyses were approved by the Institutional Review Board of the Kyungpook National University Chilgok Hospital after obtaining informed consent from the patient’s parents. Genomic DNA was extracted from peripheral blood, and library preparations were performed with the TruSight One Sequencing Panel (Illumina, Inc., San Diego, CA, USA), which enriches a 12 Mb region spanning 4813 genes of clinical relevance. Large-scale parallel sequencing was performed with the Illumina NextSeq platform. Sequence reads were aligned with the hg19 reference sequence using the Burrow-Wheeler Aligner (version 0.7.12, MEM algorithm). Duplicate reads were removed using Picard Tools (version 1.96). Local realignment and base quality recalibration were performed with the Genome Analysis Toolkit (GATK, version 3.5), and variant calling was performed using the GATK HaplotypeCaller. Variants were annotated with the Variant Effect Predictor and AnnoVar. Common variants with minor allele frequencies (≥1%) were filtered out using public databases (1000 Genomes Project, Exome Variant Server, and Exome Aggregation Consortium). Population-specific common variants were further filtered using the Korean Reference Genome Database [14]. The patient had the nonsense mutation of c.8200C > T, p(Arg2734*) in exon 32 in the *KMT2D* gene. As neither parent had this mutation, as determined by Sanger sequencing, the patient had a de novo heterozygous mutation in the *KMT2D* gene (Additional file 1: Figure S1).

We then performed additional examinations to detect other anomalies associated with Kabuki syndrome. Two-dimensional echocardiography, abdominal ultrasonography, and brain magnetic resonance imaging that had been performed at 1 year of age, showed no abnormalities. Ophthalmological findings were also normal.

The patient exhibited several endocrine problems (transiently congenital hypothyroidism, CDP, obesity and GH deficiency); additional endocrine tests were performed upon admission to our hospital (at the age of 11 years and 7 months).

The patient had transiently congenital hypothyroidism. When she was 7 days old, her free thyroxine (T4) was 1.09 ng/dL (reference range [RR], 0.8–2.0 ng/dL), triiodothyronine (T3) was 1.3 ng/mL (RR, 0.2–2.0 ng/mL), and thyroid-stimulating hormone (TSH) was 22.8 mIU/L (RR, 0.7–13.1 mIU/L). She received levothyroxine until she was 3 years old, and normal thyroid function was confirmed after discontinuing the medication. Upon admission to our hospital, she was 11 years and 7 month old her free T4 was 1.6 ng/dL (RR, 0.8–1.8 ng/dL), T3 was 0.8 ng/mL (RR, 0.6–1.9 ng/mL), and TSH was 3.5 mIU/L (RR, 0.3–4.0 mIU/L), indicating normal thyroid function.

Regarding puberty, the patient showed Tanner stage I–II of breast development; her basal luteinizing hormone level was < 0.07 mIU/mL (RR, 5–20 mIU/mL), basal follicle-stimulating hormone level was 6.19 mIU/mL (RR, 0.3–10 mIU/mL), and estradiol level was < 11.8 pg/mL (RR, 0–16 pg/mL), suggesting a CDP.

She was obese and had a buffalo hump. Her cortisol levels while awake at 8 AM, 5 PM, and midnight were 6.93 μg/dL (RR, 3–22 μg/dL), 12.46 μg/dL (RR, 3–22 μg/dL), and 14.87 μg/dL (RR, < 7.5 μg/dL), respectively. Her corresponding adrenocorticotropic hormone levels were 34.2 pg/mL, 40 pg/mL, and 42.8 pg/mL, respectively (RR for all, 0–60 pg/mL). Her urine-free cortisol was 28.9 μg/dL (RR, 4.3–176 μg/dL). Even though the midnight cortisol level was elevated, a low-dose dexamethasone suppression test showed a cortisol level of 0.68 μg/dL (RR, < 1.8 μg/dL). A lipid profile showed a total cholesterol level of 150 mg/dL (RR, 125–200 mg/dL), triglyceride level of 66 mg/dL (RR, < 200 mg/dL), and LDL-cholesterol level of 53 mg/dL (RR, < 130 mg/dL). Her aspartate aminotransferase and alanine aminotransferase levels were 24 U/L (RR, < 97 U/L) and 17 U/L (RR, < 41 U/L), respectively.

A GH stimulation test (spanning 2 days) was performed because of her short stature and delayed bone age. On the first day, the patient was administered levodopa (500 mg); the samples for the GH assessment were acquired at 0, 30, 60, 90, and 120 min. On the next day, the patient fasted for 8 h in the morning and was intravenously administered 0.1 U/kg of rapid-acting insulin diluted in 5 mL of normal saline for over 1 min, after which the samples for GH assessment were acquired at 0, 15, 30, 60, 90, and 120 min. The maximum GH concentrations in both tests were below 5 μg/L (RR, > 7 μg/L); therefore, the patient was diagnosed with GH deficiency. Her insulin-like growth factor 1 (IGF-1) and insulin-like growth factor-binding protein 3 (IGFBP-3) levels were 130.5 ng/mL (RR, 99–537 ng/mL, SDS: − 1.67) and 3760 ng/mL (RR, 2400–8400 ng/mL, SDS: 0.91), respectively. The patient started GH replacement therapy (Eutropin 0.03 mg/kg/day) when she was 11 years and 7 months old and is currently scheduled for regular follow-ups for growth and puberty changes.

## Discussion and conclusions

In the present case, a de novo nonsense mutation in the *KMT2D* gene was confirmed in a girl who had the facial features distinctive of Kabuki syndrome and several endocrine conditions such as transiently congenital hypothyroidism, CDP, obesity, and GH deficiency.

Aimée et al. first reported patients with Kabuki syndrome due to the nonsense mutation c.8200C > T, p (Arg2734 *) in exon 32 of the *KMT2D* gene in 2011 [15]. A mutation in the same position was reported by Dina et al. in patients with Kabuki syndrome and a short stature [16]. In our case, multiple endocrine conditions, including a short stature, were also observed.

KMT2D (MLL2) plays a critical role in regulating *HOX* genes and embryonic growth; it also interacts with nuclear receptors and is involved in the hormone-dependent regulation of genes that play crucial roles in reproduction and organogenesis [17]. In particular, *HOXC6* is critical for mammary gland development and milk production and plays a critical role in breast carcinoma development. Moreover, in an in vivo study, *HOXC6* was reported to regulate the expression of the bone morphogenic protein 7, fibroblast growth factor receptor 2, and IGFBP3 [18, 19]. Ansari et al. reported that *KMT2D* (*MLL2*) knockdown suppressed estradiol-medicated-*HOXC6* regulation [20]. Thus, it can be speculated that the skull anomalies, cleft palate, and short stature that are characteristic of patients with a *KMT2D* mutation are the result of FGFR2 and IGFBP3 expression via downregulation of the *HOXC6* gene. Approximately 41% of patients with Kabuki syndrome show premature thelarche, and some experience precocious puberty; these manifestations could be secondary to a low hypothalamic sensitivity to the suppressive effects of sex hormones on gonadotropin secretion [21, 22]. Our patient exhibited a CDP; this may be attributable to *HOXC6* downregulation because of the mutation in *KMT2D*, which hinders mammary gland development.

More than 50% of patients with Kabuki syndrome are overweight or obese, like our patient, during childhood or adolescence, although the exact cause is unknown [23]. The ER is associated with human disease, particularly with obesity [24]. In an in vivo study by Ohlsson et al., *ER* knockout mice had an obvious increase in total body fat [25]. Because KMT2D (MLL2) is involved in ER-dependent gene regulation, it is feasible that a mutation in *KMT2D* may be linked to obesity.

Patients with Kabuki syndrome also show postnatal growth retardation, but the cause is unclear. Schott et al. previously found that 27.8% of patients with Kabuki syndrome who were confirmed to have a *KMT2D* mutation had GH deficiency [26]. Considering that the prevalence of GH deficiency is only 1% in the general population, it is evidently very high in patients with Kabuki syndrome. The same group later showed that GH treatment produced a significant increase in height SDS after 1 year [27]. Our patient is currently receiving GH therapy because of her GH deficiency, and she is scheduled for follow-up visits to assess her growth progress.

In conclusion, a Korean girl was diagnosed with Kabuki syndrome caused by the de novo nonsense mutation of c.8200C > T, p(Arg2734*) in exon 32 in the *KMT2D* gene. The patient presented with the typical Kabuki syndrome phenotypes and several endocrine symptoms (CDP, obesity, transiently congenital hypothyroidism, and GH deficiency). Further molecular studies of the *KMT2D* gene and *HOXC6*-mediated ER regulation are required to better understand Kabuki syndrome with multiple endocrine disorders.

## Additional file

Additional file 1: **Figure S1.** Sanger sequencing confirmed a heterozygous mutation in *KMT2D*. The de novo heterozygous mutation c.8200C > T, p(Arg2734*) in exon 32 in *KMT2D* was identified by targeted exome sequencing and confirmed by Sanger sequencing analysis. (PDF 73 kb)

## Abbreviations

BMI: Body mass index
CDP: Constitutional delay of puberty
ER: Estrogen receptor
GH: Growth hormone
H3K4: Histone H3 lysine 4
IGF-1: Insulin-like growth factor-1
IGFBP-3: Insulin-like growth factor-binding protein 3
KMT2D: Histone-lysine N-methyltransferase 2D;
MLL: Mixed lineage leukemia
RR: Reference range
SDS: Standard deviation score
T3: Triiodothyronine
T4: Thyroxine
TSH: Thyroid-stimulating hormone
