# Supplementary figures and images for: A de novo KMT2D mutation in a girl with Kabuki syndrome associated with endocrine symptoms: a case report
Source: BMC Med Genet. 2018 Jun 18;19:102. doi: 10.1186/s12881-018-0606-9 (PMC6007063; doi:10.1186/s12881-018-0606-9)

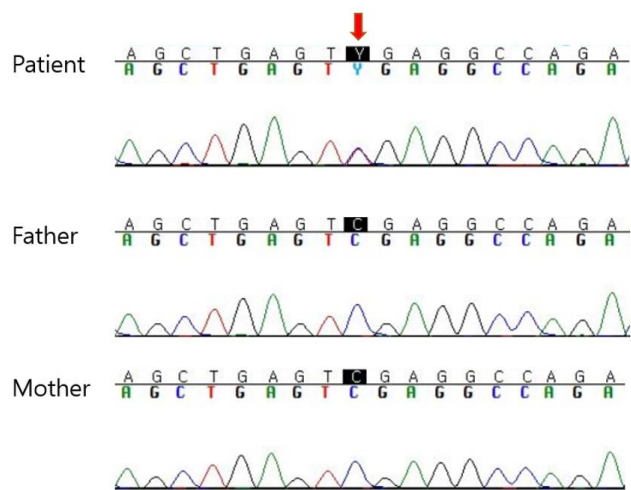

Supplement: Supplementary file 1 — Figure S1. Sanger sequencing confirmed a heterozygous mutation in KMT2D. The de novo heterozygous mutation c.8200C > T, p(Arg2734*) in exon 32 in KMT2D was identified by targeted exome sequencing and confirmed by Sanger sequencing analysis. (PDF 73 kb) [file 12881_2018_606_MOESM1_ESM.pdf]
